# Influence of Different Warm Mix Additives on Characteristics of Warm Mix Asphalt

**DOI:** 10.3390/ma14133534

**Published:** 2021-06-24

**Authors:** Anda Ligia Belc, Erdem Coleri, Florin Belc, Ciprian Costescu

**Affiliations:** 1 Faculty of Civil Engineering, Politehnica University Timisoara, 300006 Timisoara, Romania; florin.belc@upt.ro (F.B.); ciprian.costescu@upt.ro (C.C.); 2 School of Civil and Construction Engineering, Oregon State University, Corvallis, OR 97331, USA; Erdem.Coleri@oregonstate.edu

**Keywords:** warm mix asphalt, bitumen, organic additive, chemical additive, synthetic zeolite, Marshall test, stiffness modulus, permanent deformation, tensile strength ratio, water sensitivity

## Abstract

The interest in minimising fuel consumption and greenhouse gas emissions among road specialists is increasing. Thus, methods for reducing asphalt concrete mixing and compaction temperatures by a few tens of degrees Celsius without compromising the long-term performance has become a topic of significant interest. This study is focused on the analysis of warm mix asphalt (WMA) prepared with locally available materials in order to determine the suitable technology applicable to the specific traffic and climatic conditions of Romania. WMA was prepared using different warm mix additives (organic additives, chemical additive, and synthetic zeolite) at different mixing and compaction temperatures, and bitumen blends with these additives were analysed by carrying out the dynamic shear rheometer test and evaluating the penetration index. In conclusion it was noted that most additives did not lead to a significant change of bitumen’s characteristics, but the organic additive had a big influence on the bitumen’s properties. The characteristics of WMA are very similar to those of HMA. The mixing and compaction temperatures could be reduced by approximately 40 °C when WMA was blended with the additives without compromising the performance of the asphalt mixture, compared to hot mix asphalt.

## 1. Introduction

The concept of sustainable development supports the reduced consumption of raw natural materials, which increases costs and requires additional energy for production, transportation, and construction. In addition, the practice of sustainable development helps to minimise the emission of greenhouse gases and encourages the use of recycled materials without compromising the standards set by government agencies. The Brundtland Commission defines sustainable development as “the development that meets the needs of the present generation without compromising the ability of future generations to meet their own needs” [1]. Sustainable development is not only focused on the environment but also includes three pillars: economic development, social progress, and environmental protection. In all situations, technical–economic solutions that facilitate the synergy of these three pillars should be adopted so that they correspond to the “sustainable” domain.

Classic hot mix asphalt (HMA) is prepared by heating the constituent materials (aggregates and bitumen) at 160–180 °C [2,3], which significantly increases fuel consumption and greenhouse gas emissions released into the atmosphere during the manufacturing, transportation, and construction processes. The high temperatures of HMA also create safety issues for construction workers. Hence, cold asphalt mixtures are prepared at ambient temperatures using asphaltic emulsions to solve these problems. Unfortunately, cold asphalt mixtures have lower cracking and deformation performance than HMA and are not typically used for fabricating surface layers with high durability.

Warm mix asphalt (WMA) has been used worldwide to combine the advantages of HMA and cold mix asphalt [4,5]. WMA mixtures are prepared and compacted at significantly lower temperatures than HMA mixtures (mixing temperatures of approximately 120–150 °C compared to 160–180 °C for HMA, and compaction temperatures of approximately 100–130 °C compared to 150–170 °C for HMA). The required workability for adequate mixing and compaction is achieved using additives (chemical or organic) or zeolites (natural or synthetic). According to a study, the application of these techniques can reduce greenhouse gas emissions by 10–50% and fuel consumption by 11–35% [4]. Furthermore, the WMA production requires about 30% less energy than HMA [6]. For further reducing the environmental impact of the WMA, the noise level generated by the interaction between the road pavement and the car tire can be significantly reduced by applying a microsurfacing [7].

According to [8], the techniques used for producing WMA mixes can be classified as follows: foaming, organic additive, and chemical additive techniques. Various foaming techniques have been mentioned in the literature, such as water containing additives [9,10,11] and water based processes [12,13]. Hydrated lime is also used more and more as an additive [14]. These techniques are aimed to reduce binder viscosity during the mixing and compaction of the asphalt mixture. Several methods are used for adding small quantities of water into hot binders [15]. When water is mixed with bitumen, the water transforms into vapour, which increases the binder volume and decreases the binder viscosity for a limited time [16]. This behaviour signifies that when the mixture cools, and the formed foam disappears, the bitumen starts to exhibit the characteristics of virgin bitumen. This foaming process facilitates uniform blending during asphalt mixture production. In addition, the aggregates are more uniformly coated with the asphalt binder owing to this process. A study evaluated the effect of foaming water on the bitumen’s properties and it was found that the rheological properties are greatly influenced, but it lead to an improved temperature sensitivity [17]. Another study revealed that the type of zeolite is influencing the efficiency of the binder foaming [18]. Another research team concluded that the foaming based technologies lead to a lower moisture resistance [19]. The main effects of the use of zeolites in WMA are a decrease of the compaction temperature of the WMA, and improved coating and workability. The main problems reported are related to a higher moisture damage and a lower resistance to permanent deformation. Natural zeolites are considered to be viable replacements for synthetic zeolites [20].

Organic additives are directly added to the bitumen or mixed with the asphalt mixture to reduce the bitumen viscosity. The reduced binder viscosity increases the compactability, even at lower temperatures. Organic additives must be selected such that their melting temperature exceeds the temperature expected for the bituminous layer to reach the field for it to have lower susceptibility to low temperatures and exhibit good performance at permanent deformations [21]. Some advantages of using the organic additives are better or equal performance to that of HMA at lower temperatures and increased rutting resistance [22,23]. Another advantage is a better high temperature performance when used in percentages up to 2% by the weight of the bitumen. One negative effect of the organic additive is the decreased low temperature performance [24]. Research [25] compared the stiffness and shear fatigue of HMA and WMA with organic additive concluding that WMA had an increased stiffness and a similar fatigue behaviour compared to HMA. Different opinions are related to the addition of synthetic wax to a polymer modified bitumen, some stating that it can lead to a lower resistance to low temperature cracking [26], while others consider it safe to be used due to the fact that the increase in the cracking temperature is not significant [27].

The chemical additives do not influence the viscosity of the bitumen; instead, they act as surfactants and reduce the friction at the binder–aggregate interface. Thus, a more uniform blend between the binder and aggregates can be achieved during the plant mixing process, and the asphalt mixture can be compacted at lower temperatures [28]. Using a chemical additive in a WMA can lower the production temperatures, improve compactibility of the mixtures and decrease the rutting potential [29]. It was found that WMA with chemical additives might have a lower Marshall stability than HMA [30]. The addition of a chemical additive can lower the air voids, but it may increase the potential for moisture damage [31].

The advantages of the WMA can be summarised as follows:

- improvement in working conditions for workers owing to minimised emissions and exposure to less heated work environments;
- reduction in the emission of greenhouse gases, which are harmful to the environment;
- ability to lay bituminous layers at lower temperatures, which results in an increased construction period;
- longer haulage distances;
- easier compaction owing to improved workability;
- quicker commissioning of the bituminous layer for traffic use;
- possibility of adding large quantities of reclaimed asphalt pavement without compromising performance;
- reduction in total energy consumption during the production of bituminous layers [32,33,34,35].

In Europe, based on laboratory and field pavement performance data (data obtained within the last four years), WMA mixes appear to exhibit similar or even better performance than HMA mixes [36,37,38,39]. Additionally, similar performances have been reported in the USA between the two asphalt mixture types after two years of use [40]. However, the long-term performance of WMA mixes has not been investigated in detail owing to limited field data. Based on previous research studies, some aspects of WMA mixes, such as water sensitivity, stiffness, and resistance to permanent deformation, need to be examined.

In this study, WMA mixtures were prepared using two types of organic additives (a synthetic wax (W1) and a softer synthetic wax (W2)), a chemical additive (C), and a synthetic zeolite additive (Z). Additionally, the behaviour of virgin bitumen and bitumen blended with the mentioned additives were evaluated and were correlated to the results obtained on the WMA mixes. The main purpose is to determine the optimum mixing and compaction temperatures for conditions and materials peculiar to Romania.

The specific objectives of this study are as follows:

- to quantify the effect of additives on the performance of WMA mixtures;
- to evaluate the physical–mechanical characteristics of the different types of WMA;
- to determine the optimum mixing and compaction temperatures suitable for WMA;
- to select the optimum WMA suitable for the climatic and traffic conditions peculiar to Romania.

The production of WMA is still little promoted by some road administrations. This is also the case of Romania, which does not have technical norms for the production of these asphalt mixtures, and their application remains completely isolated. In this context, the research provides results that confirm that WMA can be obtained with performances comparable to HMA by using locally available materials and by applying the Romanian technical norms for HMA. The results offer the chance of comparing the performances obtained in the case of using different warm mix additives, both in terms of the high temperature behaviour of a certain bitumen (by DSR test) and the physical–mechanical characteristics of a certain type of WMA, highlighting the particularities for each additive.

## 2. Materials and Methods

### 2.1. Materials

**Figure 1:**
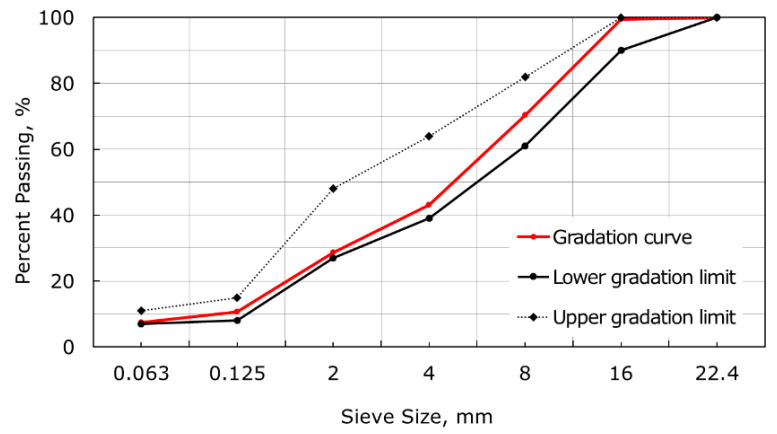
The gradation curve for the asphalt mixture.

This section provides details on the virgin binder, virgin aggregates, and additives used in this study. Asphalt concrete for the surface layer was prepared using natural aggregate with a nominal maximum aggregate size (NMAS) of 16 mm. The asphalt concrete consisted of natural crushed aggregates crushed aggregates (25.6% crushed stone 4/8 mm, 28.4% crushed stone 8/16 mm, and 25.6% crushed sand 0/4 mm) obtained from diorite, 7.6% natural sand (0/4 mm), 7.6% filler, and 5.2% virgin binder with 50/70 penetration grade. All percentages are given by weight of the asphalt mixture. The gradation curve is presented in Figure 1. The binder content of the asphalt mixture was 5.2% of the total weight of the mixture. The following materials were used to prepare the WMA mixtures: two types of organic additives (a synthetic wax (W1) and a softer synthetic wax (W2)), a chemical additive (C), and a synthetic zeolite (Z).

The bitumen used for the laboratory tests was 50/70 penetration grade virgin bitumen, which is frequently used in the construction of Romanian roads. In addition, the bitumen was blended with various substances used in road construction to reduce the mixing and compaction temperatures of asphalt mixtures, i.e., to obtain WMA mixtures. In Europe, bitumen is graded based on the minimum and maximum penetration values at 25 °C. In the USA, it is graded based on the minimum and maximum pavement temperatures.

The organic additives used in this study are synthetic waxes which are produced using the Fischer–Tropsch process. They are a mix of long chain hydrocarbons obtained from coal gasification. The second organic additive, W2, is a softer synthetic wax, having a melting temperature of about 85 °C compared to the first organic additive, W1, which has a melting temperature of about 115 °C.

The chemical additive is a viscose liquid and is composed of amino substance derivatives.

The synthetic zeolite used is a manufactured sodium aluminium silicate which has been hydro-thermally crystallized. It contains an amount of 21% crystalline water by mass which is released in the temperature range of 85–180 °C.

### 2.2. Experimental Plan

#### 2.2.1. Bitumen Tests

This part of the study was focused on determining the characteristics of virgin bitumen and how additives influence these characteristics. Therefore, the following tests were performed: penetration tests at 25 °C [41], softening point ring and ball method tests [42], and ductility tests [43]. In addition, the penetration index for each case was calculated.

The main laboratory test that indicates the bitumen type is the standard penetration at 25 °C, which indicates the bitumen viscosity in the solid state. Generally, the softer the bitumen, the higher the penetration value.

The softening point ring and ball method is the main laboratory technique used for determining the plasticity of bitumen (plasticity is the property of consistent materials to deform permanently without cracking under the action of loads).

The penetration index PI is a measure of the thermal sensitivity of bitumen, and it is calculated using an equation based on the known standard penetration value at 25 °C and the softening point. The penetration index is a dimensionless parameter. It should be noted that for the 50/70 penetration grade virgin bitumen, the penetration index must satisfy the relationship: −1.5 < PI < 0.7. The lower the penetration index, the more rapid the binder consistency varies with temperature. The penetration index is expressed in Annex A of reference [44] as follows:

PI = [20 × t_R&B_ + 500 × lg P − 1952]/[t_R&B_ − 50 × lg P + 120] (1)

where PI is the penetration index (−); t_R&B_ is the softening point—ring and ball method (°C); lg P is the logarithm of standard penetration at 25 °C (0.1 mm).

A dynamic shear rheometer (DSR) was used to characterise the viscoelastic behaviour of bitumen at medium and high temperatures. This equipment was used to determine the risk of rutting onset and fatigue cracking resistance [45,46,47,48]. For the DSR test, a thin bitumen sample was positioned between two 25 mm circular plates. The bottom plate was secured in position, and the top plate oscillated back and forth on the sample at an angular frequency of 10 rad/s, which corresponded to a traffic speed of 90 km/h, to induce shearing [49,50]. The test temperatures ranged from 25 to 85 °C at 10 °C increments, and for each temperature, the test frequencies ranged from 0.1 to 10 Hz, which represented 10 frequencies at equal spacings of 10 logarithmic steps. The complex shear modulus G* and phase angle φ were obtained from the test results. For each temperature, 10 values of the complex shear modulus and phase angle, which corresponded to the 10 loading frequencies, were recorded. The bitumen behaviour is more elastic at low phase angles (purely elastic at φ = 0 °C) and more viscous at high phase angles (purely viscous at φ = 90 °C).

The evaluated bitumen blends were as follows: virgin bitumen, bitumen with 3% synthetic wax by binder weight (Bitumen+3%W1), bitumen with 1.5% synthetic wax by binder weight (Bitumen+1.5%W1), bitumen with 1.5% softer wax by binder weight (Bitumen+1.5%W2), bitumen with 0.5% chemical additive by binder weight (Bitumen+0.5%C), and bitumen with 5.5% synthetic zeolite by binder weight (Bitumen+5.5%Z). The percentages of additives and synthetic zeolite were a recommendation from suppliers. The blend between the virgin bitumen and the additive was made with a countertop electric laboratory stirrer that can reach up to 3000 revolutions per minute.

#### 2.2.2. Asphalt Mixture Tests

In this part of the study, the long-term performance of different types of asphalt mixtures was evaluated by comparing the test results. The evaluated asphalt mixtures were as follows: HMA, asphalt mixture mixed and compacted at WMA temperatures (HMA_LT), WMA containing 3% wax by binder weight (WMA_3%W1), WMA containing 1.5% wax by binder weight (WMA_1.5%W1), WMA containing 1.5% softer wax by binder weight (WMA_1.5%W2), WMA containing 0.5% chemical additive by binder weight (WMA_0.5%C), and WMA containing 0.3% synthetic zeolite by the weight of the mixture (WMA_0.3%Z). All specimen followed the same mixture design, with only additives different. The percentages of additives and synthetic zeolite were a recommendation from suppliers. For all the asphalt mixtures, Marshall stability and flow [51], stiffness modulus [52], resistance to permanent deformation (creep characteristics) [53], and water sensitivity [54,55] were determined. Table 1 lists the experimental plan adopted in this study.

The preparation of laboratory-mixed laboratory-compacted (LMLC) specimens: virgin aggregates and bitumen were used to prepare the specimens. The specimens were mixed in the laboratory using a mechanical mixer. The Marshall specimens were compacted according to [56], while the specimens with the gyratory press were compacted according to [57].

For HMA preparation, the mixing and compaction temperatures were 160 and 150 °C, respectively. The temperatures listed in Table 2 were used for the mixing and compaction of WMA.

In this study, two types of specimens were used:

- Marshall specimens, which were compacted to 50 blows on each side (fixed compactive effort) using a Marshall hammer;
- specimens compacted using a gyratory compactor at 80 gyrations (to determine the stiffness modulus and resistance to permanent deformation) and 50 gyrations (to determine the water sensitivity).

### 2.3. Test Methods

The test methods adopted in this study were the Marshall test, the test applying indirect tension to cylindrical specimens (IT-CY) to determine the stiffness modulus, triaxial cyclic compression test to evaluate the resistance to permanent deformation, and indirect tensile test using the tensile strength ratio (TSR) to determine the water sensitivity.

#### 2.3.1. Marshall Test

Marshall tests were conducted to determine the resistance of 100 mm diameter cylindrical specimens of asphalt mixtures to plastic flow. The thickness of the samples was 63.5 ± 3 mm. The tests were performed at 60 °C. Marshall stability and flow were the two significant properties determined through the Marshall tests. The stability indicated the maximum load supported by the specimen at the deformation rate, whereas the flow represented a measure of the deformation of the specimen owing to loading and was determined in relation to the maximum recorded load.

Marshall stability and flow were used to evaluate the asphalt mixtures for laboratory mix design. The Romanian standard [58] specifies strict requirements for the minimum and maximum values for Marshall stability and flow.

#### 2.3.2. Stiffness—Test Applying Indirect Tension to Cylindrical Specimens (IT-CY)

The stiffness of the asphalt mixtures was evaluated through indirect tensile tests on cylindrical specimens (IT-CY). The stress–strain response of the pavement under traffic loads was analysed. The tests were performed at 20 °C. Each specimen had a diameter of 100 mm and a thickness of approximately 65 mm, and it was prepared using a gyratory compactor. The Poisson’s ratio was assumed to be 0.35.

Through the tests, the stiffness modulus of the asphalt mixtures was determined. The Romanian code [58] recommends a minimum value, in which the stiffness modulus must be reached to ensure that the asphalt mixture achieves an adequate performance level.

#### 2.3.3. Resistance to Permanent Deformation—Triaxial Cyclic Compression Test

Triaxial cyclic compression tests with confinement were performed to assess the resistance to permanent deformation (creep characteristics). The tests were performed at 50 °C. Each specimen had a diameter of 100 mm and a thickness of approximately 65 mm and prepared using the gyratory compactor.

The deformation and creep rate of the asphalt mixture samples were calculated using the test results. The test results were used to evaluate the asphalt mixture performance and design new types of asphalt mixtures. The Romanian standard [58] specifies maximum values for the deformation and creep rate to achieve an adequate performance level for asphalt mixtures.

#### 2.3.4. Water Sensitivity—Indirect Tensile Test

Several tests are used for analysing the moisture susceptibility of asphalt mixtures [59,60,61,62]. In this study, indirect tensile tests were performed on conditioned and unconditioned specimens to determine the water sensitivity of asphalt mixtures. Six samples of the asphalt mixtures (three conditioned and three unconditioned samples), each with a diameter of 100 mm, were tested. The specimens were compacted at 50 gyrations using the gyratory press, according to specifications [54].

The TSR was calculated using Equation (1) [54]:

TSR = S_w_/S_d_ × 100 (2)

where TSR is the tensile strength ratio (%); S_w_ is the average tensile strength of the conditioned (wet) specimens (kPa); S_d_ is the average tensile strength of the unconditioned (dry) specimens (kPa).

## 3. Results and Discussion

### 3.1. Bitumen Test Results

#### 3.1.1. Penetration Index

Bitumen facilitates the cohesion between aggregates and fillers within asphalt mixtures, and its mechanical and rheological characteristics significantly influence the behaviour and durability of bituminous layers. The rheological behaviour of bitumen directly controls the behaviour of asphalt mixtures in service. Soft bitumen is susceptible to high temperatures and can cause the large plastic deformation of bituminous layers, whereas stiff bitumen can induce the cracking of bituminous layers.

**Figure 2:**
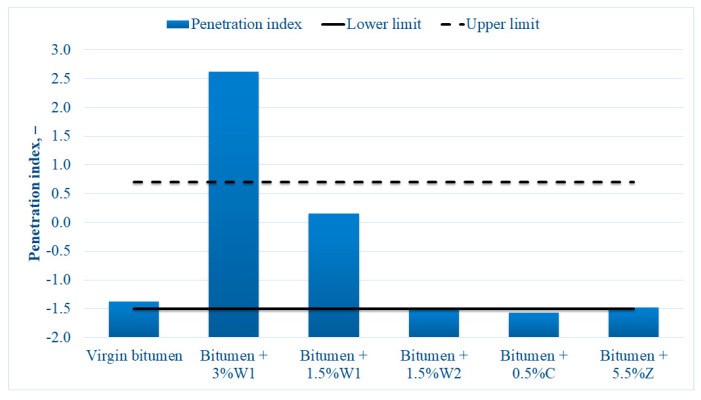
Penetration index values for different bitumen blends.

The penetration index results are shown in Figure 2.

It was found that the addition of additives to the bitumen to decrease the mixing temperature of the asphalt mixtures influenced the basic characteristics of the binder. The wax (W1) tended to make bitumen less susceptible to temperature, whereas the other additives caused the binder to be more susceptible to temperature. A high percentage (3%) of the wax generated the most significant change in the characteristics of the 50/70 penetration grade virgin bitumen. However, the addition of the other additives did not significantly alter these characteristics, and the penetration index values were within the range recommended for 50/70 penetration grade virgin bitumen [63].

#### 3.1.2. Dynamic Shear Rheometer Test

For the six bitumen blends, the complex shear modulus and phase angle were determined using the DSR. It was observed that the complex shear modulus decreased with increasing temperature but increased with increasing frequency. Conversely, the phase angle increased with increasing temperature but decreased with increasing frequency.

**Figure 3:**
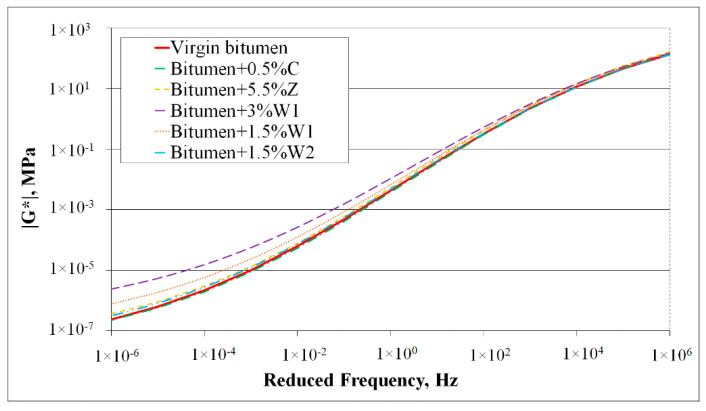
DSR master curves for complex modulus |G*| of 50/70 virgin bitumen and bitumen blends containing additives.

Figure 3 shows the master curves for the complex shear modulus of the virgin bitumen and five bitumen blends containing additives. The master curves were plotted, considering the reference temperature of 55 °C. It was observed that for bitumen blended with a chemical additive (C) or a synthetic zeolite (Z), its behaviour was similar to that of virgin bitumen (Figure 2). Nevertheless, the complex shear modulus of the bitumen slightly increased when synthetic zeolite was added.

For bitumen containing organic additives, a different phenomenon was observed. Although the behaviour of bitumen containing the softer wax (W2) was similar to that of the virgin bitumen, the behaviour of the bitumen blended with both percentages of the classic wax (W1) significantly differed from the virgin bitumen behaviour. At low frequencies, a considerable increase in the complex shear modulus occurred, which was most significant when the 3% organic additive (W1) was used.

**Figure 4:**
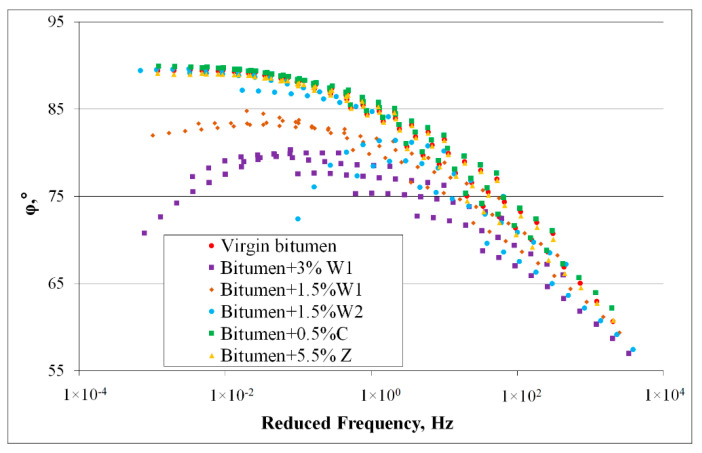
DSR master curves for phase angle φ of 50/70 virgin bitumen and bitumen blends containing additives.

Figure 4 shows the effects of temperature and frequency on the complex shear moduli of all the bitumen blends mentioned above.

The phase angles of the bitumen blended with the chemical additive (C) and synthetic zeolite (Z) respectively were almost identical to those of the virgin bitumen (Figure 3). Hence, it was inferred that the addition of these additives did not modify the rheology of the bitumen. The results were entirely different when organic additives were used. The phase angles and trends for bitumen that contained organic additives were significantly different from those of the virgin bitumen, particularly at high temperatures (55−85 °C). This result indicated that some changes in the bitumen properties occurred with the addition of organic additives. Moreover, the maximum difference was observed for bitumen blended with the 3% wax (W1), which demonstrated that the addition of 3% wax (W1) significantly influenced bitumen behaviour.

**Figure 5:**
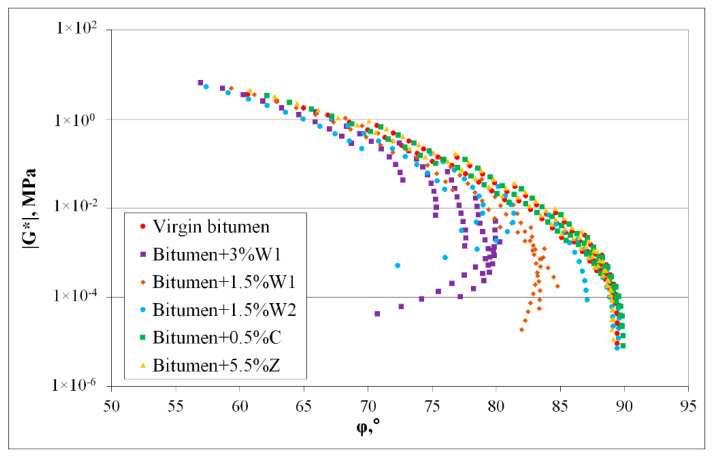
Variations of complex shear modulus with phase angle (Black diagram) for 50/70 virgin bitumen and bitumen blends containing additives.

Figure 5 (Black diagram) depicts the variations of the complex shear modulus with the phase angle at all considered temperatures for both the virgin bitumen and the bitumen blended with additives.

The values and curve shapes for both the virgin bitumen and bitumen blends with chemical additive (C) and synthetic zeolite (Z), respectively, were similar. This behaviour proved that the bitumen was not adversely affected by the addition of the chemical additive or synthetic zeolite. A significant difference between the values measured for the virgin bitumen and those for the bitumen blended with organic additives was observed. The bitumen blended with the softer wax (W2) showed a significant difference at 55−65 °C. However, for the bitumen blended with classic wax (W1), differences were observed at almost all temperatures.

These conclusions correlated with the results of the classical tests on bitumen blends. Similar to the penetration index, bitumen hardening was observed with the addition of wax during the DSR tests. Moreover, the same properties were preserved when bitumen was blended with synthetic zeolite (Z) or chemical additive (C).

### 3.2. Asphalt Mixture Test Results

#### 3.2.1. Marshall Test

Marshall stability is used to determine the optimum composition of asphalt mixtures and analyse the behaviour of the bituminous layer under traffic loads at high temperatures. The flow index, which is determined simultaneously with stability, is the deformation reached by the vertical diameter of the specimen at failure and is expressed in millimetres. A low Marshall stability (below the standard limit) is generally supplemented with a high Marshall flow (higher than 4.5–5.0 mm); it indicates that the asphalt mix has low stability at high temperatures (e.g., bitumen with excess or reduced consistency, having a weak aggregate skeleton, or containing excess sand). A very high Marshall stability, in correlation with a low Marshall flow (below 1.5 mm), may indicate that the bitumen is inadequate (bitumen having very high consistency or burnt during the asphalt mix preparation).

**Figure 6:**
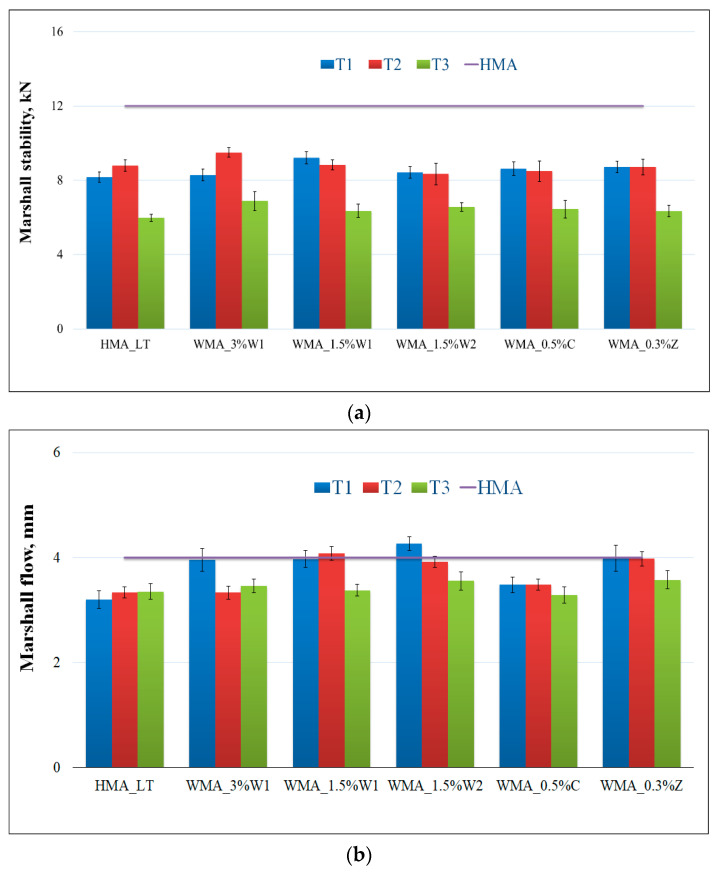

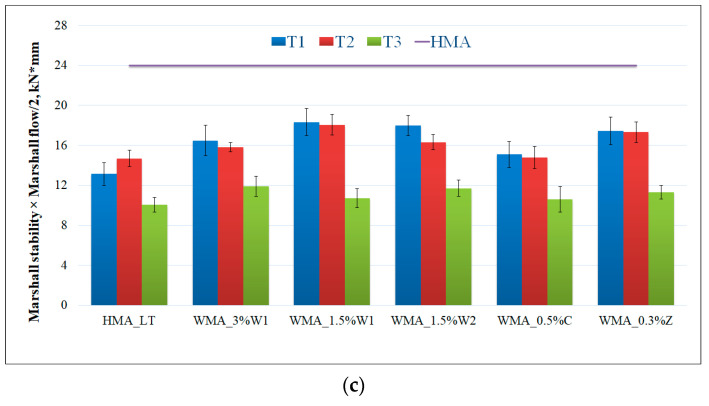
Marshall test results: (**a**) Marshall stability; (**b**) Marshall flow; (**c**) (Marshall stability × Marshall flow)/2 (length of the error bar is equal to one standard deviation).

Figure 6a,b show the values of the Marshall stability and flow for all the asphalt mixtures at different temperatures (see Table 2). In Figure 6a,b, the horizontal lines represent the values obtained for the Marshall stability and Marshall flow for HMA. Figure 6c depicts a new parameter that indicates the flexibility of the asphalt mix [64]. It was calculated using the following formula: (Marshall stability × Marshall flow)/2. Moreover, the horizontal line represents the value obtained for HMA.

The Marshall stability values for all the WMA mixtures were close, but comparatively lower than the value obtained for HMA. The same conclusion was drawn by studies in Lithuania [30,37]. It should be noted that although the WMA values were lower than the one of HMA, the WMA values are within the limits of 6.5–13 kN specified in the Romanian code [58]. The Marshall flow values were close in all the investigated cases, and no significant changes were observed. The Marshall flow values of 1.5–4 mm were also within recommended limits provided in the Romanian standard.

Regarding the new parameter considered in this study, the values for WMA in all the analysed cases were higher than those for the asphalt mixtures prepared at lower temperatures (HMA_LT); this result indicated high flexibility.

The results obtained at different temperatures were compared by using a statistical test, two-sample *t*-test. Calculated *p*-values for the statistical test are given in Table 3. Suppose F1 and F2 are two distributions. Possible hypotheses and alternatives concerning these distributions are [65]:

H_0_: F1(x) = F2(x) (3)

H_A_: F1(x) ≠ F2(x) (4)

A decision rule was adopted for the two-sample *t*-test. The decision rule is as follows:

Reject H_0_ if *p*-value < 0.05 (5)

Fail to reject H_0_ if *p*-value ≥ 0.05 (6)

If *p*-value ≥ 0.05 means the difference between the two distributions is statistically insignificant (in other words, they can be considered equal).

In all the considered cases, the differences between the mixing and compaction temperatures of 140–120 °C and 120–120 °C, respectively, were insignificant. This trivial difference indicated that 120 °C could be suitably adopted for both mixing and compaction and provides an advantage of reducing the costs of heating aggregates up to 140 °C. Mixing and compaction temperatures of 100–120 °C indicate lower values in all the cases, which suggests that a significant decrease in the compaction temperature results in the loss of stability of the asphalt mix. On the other hand, based on the given decision rule, the statistical test shows there are significant differences between T1 and T3, and T2 and T3, respectively.

#### 3.2.2. Stiffness—Test Applying Indirect Tension to Cylindrical Specimens (IT-CY)

**Figure 7:**
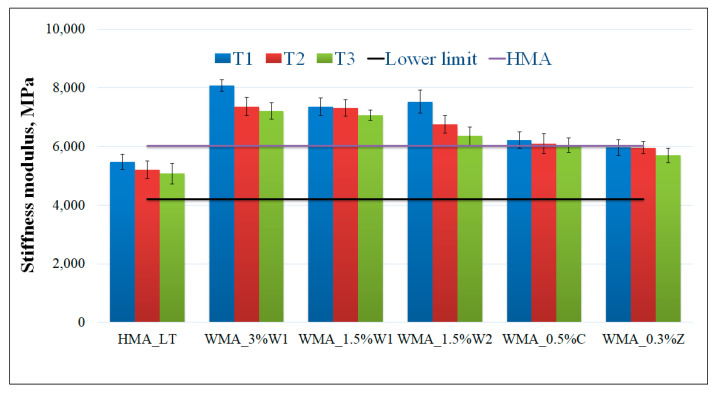
Stiffness modulus values for different asphalt mixtures (length of the error bar is equal to one standard deviation).

Figure 7 shows the results for the indirect tensile tests on 100 mm diameter cylindrical specimens. The black horizontal line represents the minimum value of the stiffness modulus specified by the Romanian standard [58], and the purple horizontal line represents the stiffness modulus value obtained for the HMA.

Some improvement was observed in WMA blended with both types of wax. At all the considered temperatures (Table 2) and for all the percentages of organic additive used, the values exceeded not only the specified minimum limit but also exceeded the HMA values. For the WMA blended with chemical additive (C) and synthetic zeolite (Z), respectively, the stiffness modulus values were the closest to those obtained for HMA, and they significantly exceeded the limit provided in the Romanian code and the values obtained for the asphalt mix prepared at low temperatures (HMA_LT).

It was found that the stiffness modulus decreased with temperature, similar findings have been reported by other scientists [38,66,67]. The increase in the stiffness modulus of WMA compared to that in the low-temperature asphalt mixtures was attributed to the addition of the additives. In the case of WMA containing synthetic wax, a similar trend for the penetration index of bitumen blended with organic additives was observed, which signified that both the bitumen and asphalt mixture became stiffer with the addition of wax.

The influence of various additives on the stiffness modulus of WMA was studied by a number of researchers. A study shows that at a mixing temperature of 120 °C the stiffness modulus for WMA, with six additives, presents higher values than WMA [66]. Another study focused on the WMA with synthetic wax also reports an increase of the stiffness moduli for WMA with wax compared to HMA [68].

In order to assess the significance of the differences between results at different temperatures the two-sample *t*-test was used. The same conditions were applied, as in Section 3.2.1. Calculated *p*-values for the statistical test are given in Table 4.

In all the considered cases, it was concluded that the differences between the mixing and compaction temperatures of 140–120 °C, 120–120 °C, 120–100 °C, respectively, were insignificantly different from one another.

#### 3.2.3. Resistance to Permanent Deformation—Triaxial Cyclic Compression Test

**Figure 8:**
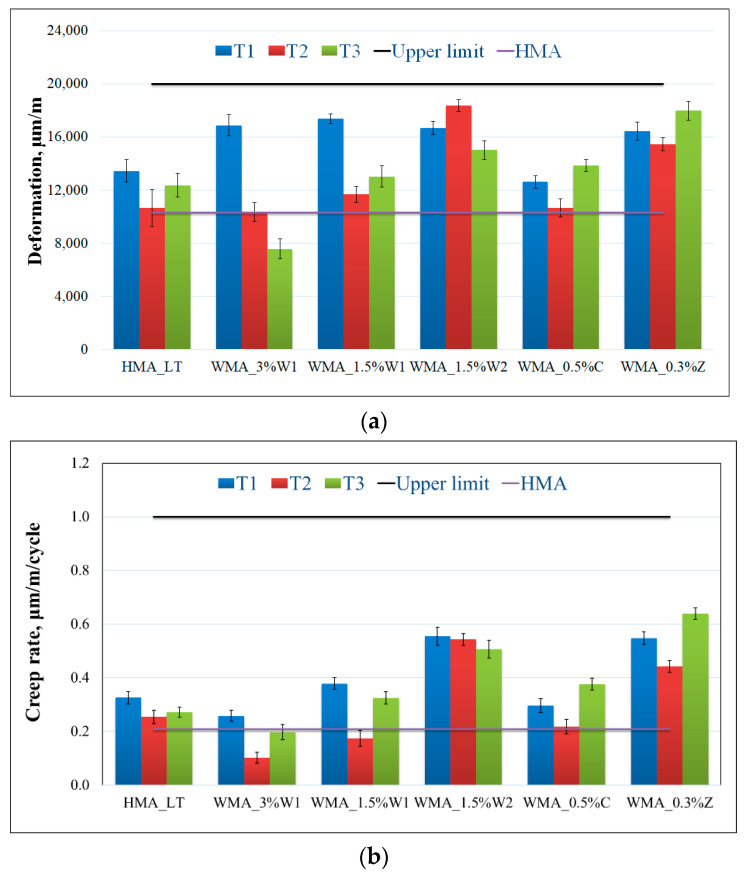
Triaxial cyclic compression test results: (**a**) deformation; (**b**) creep rate (length of the error bar is equal to one standard deviation).

Figure 8a,b show the deformation and creep rate results, respectively, for triaxial cyclic compression tests on 100 mm diameter cylindrical specimens subjected to 300 kPa and 10,000 pulses at 50 °C. In Figure 8a,b, the black horizontal line represents the maximum value for the deformation and creep rate, respectively, specified by the Romanian standard. The horizontal purple line represents the corresponding values for the deformation and creep rate of HMA.

This test is designed to evaluate the resistance to permanent deformation. It enables the classification of different types of asphalt mixtures and the acceptability verification of asphalt mixtures. However, it does not allow for the quantitative prediction of rutting of the asphalt mixtures in service.

Most of the results for all the considered temperatures (Table 2) showed a higher increase in the deformation and creep rate of WMA than HMA. However, the values for both deformation and creep rate were below the limits recommended in the Romanian standard.

A variability between the trends was observed among the WMA mixtures. However, no particular increasing or decreasing variation with temperature was observed. Increased values of deformation and creep rate could indicate a lower resistance to high temperatures, owing to lower preparation and compaction temperatures.

A two-sample *t*-test was performed between results at all mixing—compaction temperatures to determine significant differences, if any. Table 5 summarizes the results of the statistical test. The same conditions were applied, as in Section 3.2.1.

As all the *p*-values are greater than 0.05 it means the difference between the two distributions is statistically insignificant.

#### 3.2.4. Water Sensitivity—Indirect Tensile Test

**Figure 9:**
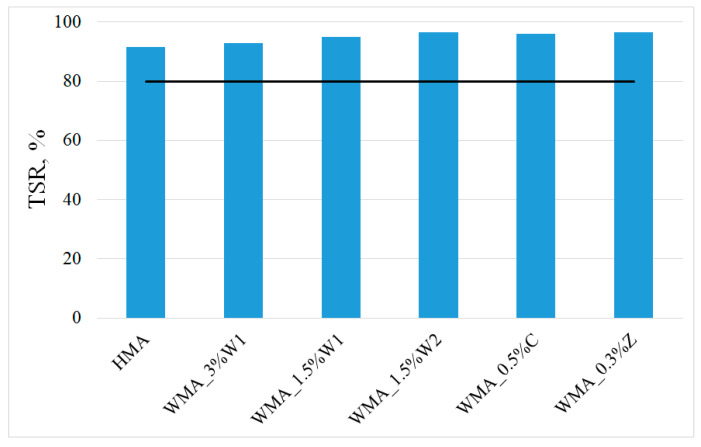
TSR values for different asphalt mixtures.

Figure 9 shows the values of the TSR, which indicates the water sensitivity of the asphalt mixture specimens. In Figure 9, the black horizontal line represents the minimum acceptable limit provided by the Romanian standard for asphalt mixtures.

It was observed that all TSR values were close, but the WMA values were slightly higher than the one for HMA, which was considered as the reference. This behaviour could be attributed to the additives used, which increased the moisture resistance and/or influenced the compaction at lower temperatures. Similar results, with a maintenance or a slight improvement of the TSR value for WMA compared to HMA, have been reported by other studies [21,66]. These close values suggested that in terms of moisture sensitivity, the WMA can be used to replace HMA successfully.

## 4. Conclusions

In Romania, studies on WMA mixtures have not yet been investigated extensively, and currently, no standards for their industrial application have been provided. This study was aimed at establishing the possibilities of using WMA for the specific traffic and climatic conditions of Romania and determining the additives that can achieve the highest performance.

In the first part of this study, it was determined whether the introduction of additives in bitumen modifies the characteristics of bitumen. Necessary tests on bitumen (penetration tests at 25 °C, softening point ring and ball method, and ductility) were performed. However, the test results on the susceptibility of bitumen to high temperatures (DSR tests) indicated that most additives did not change the characteristics of bitumen. However, the classic wax (W1) significantly modified and hardened the bitumen and caused the bitumen to be less susceptible to temperature. It was found that all the considered additives were suitable for use, with the observation that an addition of over 1.5% of wax by binder weight altered the basic characteristics of bitumen.

The second part of the study was aimed at comparing the characteristics determined in the laboratory for HMA and WMA mixed–compacted at various temperatures (140–120, 120–120, and 120–100 °C) using the additives mentioned above.

The results obtained for the WMA Marshall specimens (stability and flow) showed that WMA containing additives generally exhibited characteristics similar to those of HMA. For the dynamic tests, the WMA mixtures had higher stiffness modulus values than the HMA, except for the WMA blended with synthetic zeolite (Z). Overall, the deformation and creep rate values of WMA were higher than those of HMA but did not exceed the recommended limits.

All the results obtained in this study demonstrated that additives could be used in the production of WMA and these asphalt mixtures could be applied to the traffic and climatic conditions of Romania. The WMA containing chemical additive (C) has the closest behaviour to HMA. For all the additives used in this study, the mixing and compaction temperatures can be effectively reduced by approximately 40 °C, and the best laboratory results were obtained at a mixing and compaction temperature of 120 °C.

## Figures and Tables

**Table 1:** Experimental plan for laboratory-mixed laboratory-compacted (LMLC) samples.

Test Type	Asphalt Mixture Type	Mixing— Compaction Temperatures (°C)	Test Temperature (°C)	Replicates	Total Number of Tests
Marshall test	HMA	160–150	60	3	3
	HMA_LT WMA_3%W1 WMA_1.5%W1 WMA_1.5%W2 WMA_0.5%C WMA_0.3%Z	140–120 120–120 120–100	60	3	54
Stiffness—indirect tension to cylindrical specimens (IT-CY)	HMA	160–150	20	3	3
	HMA_LT WMA_3%W1 WMA_1.5%W1 WMA_1.5%W2 WMA_0.5%C WMA_0.3%Z	140–120 120–120 120–100	20	3	54
Resistance to permanent deformation— triaxial cyclic compression test	HMA	160–150	50	3	3
	HMA_LT WMA_3%W1 WMA_1.5%W1 WMA_1.5%W2 WMA_0.5%C WMA_0.3%Z	140–120 120–120 120–100	50	3	54
Water sensitivity—indirect tensile test	HMA	160–150	25	3	3
	HMA_LT WMA_3%W1 WMA_1.5%W1 WMA_1.5%W2 WMA_0.5%C WMA_0.3%Z	120–120	25	3	18

**Table 2:** Test temperatures for WMA specimens.

ID	Mixing Temperature (°C)	Compaction Temperature (°C)
T1	140	120
T2	120	120
T3	120	100

**Table 3:** *p*-values from two-sample *t*-test comparing Marshall test results at different temperatures.

Compared Distributions	*p*-Value		
	Marshall Stability	Marshall Flow	Marshall Stability × Marshall Flow/2
T1–T2	0.3838	0.5798	0.8018
T1–T3	0.0000	0.0674	0.0000
T2–T3	0.0000	0.1346	0.0000

**Table 4:** *p*-values from two-sample *t*-test comparing stiffness modulus results at different temperatures.

Compared Distributions	*p*-Value
	Stiffness Modulus
T1–T2	0.5652
T1–T3	0.3447
T2–T3	0.6739

**Table 5:** *p*-values from two-sample *t*-test comparing triaxial cyclic compression test results at different temperatures.

Compared Distributions	*p*-Value	
	Deformation	Creep Rate
T1–T2	0.1226	0.2565
T1–T3	0.2002	0.9325
T2–T3	0.8236	0.3318

## Data Availability

Data sharing is not applicable to this article.
